# A point mutation in the *Pdia6* gene results in loss of pancreatic β-cell identity causing overt diabetes

**DOI:** 10.1016/j.molmet.2021.101334

**Published:** 2021-09-04

**Authors:** Nirav Florian Chhabra, Anna–Lena Amend, Aimée Bastidas-Ponce, Sibylle Sabrautzki, Marta Tarquis-Medina, Stephan Sachs, Marina Rubey, Bettina Lorenz-Depiereux, Annette Feuchtinger, Mostafa Bakhti, Heiko Lickert, Gerhard K.H. Przemeck, Martin Hrabě de Angelis

**Affiliations:** 1 Helmholtz Zentrum München, Institute of Experimental Genetics and German Mouse Clinic, Neuherberg, Germany; 2 German Center for Diabetes Research (DZD), Neuherberg, Germany; 3 Helmholtz Zentrum München, Institute of Diabetes and Regeneration Research, Neuherberg, Germany; 4 Technische Universität München, School of Medicine, Munich, Germany; 5 Helmholtz Zentrum München, Research Unit Comparative Medicine, Neuherberg, Germany; 6 Helmholtz Zentrum München, Institute of Human Genetics, Neuherberg, Germany; 7 Helmholtz Zentrum München, Research Unit Analytical Pathology, Neuherberg, Germany; 8 Technische Universität München, TUM School of Life Sciences (SoLS), Chair of Experimental Genetics, Freising, Germany

**Keywords:** Pdia6, Insulin, Islets, Diabetes, ER stress

## Abstract

**Objective:**

Protein disulfide isomerases (PDIs) are oxidoreductases that are involved in catalyzing the formation and rearrangement of disulfide bonds during protein folding. One of the PDI members is the PDI-associated 6 (PDIA6) protein, which has been shown to play a vital role in β-cell dysfunction and diabetes. However, very little is known about the function of this protein in β-cells *in vivo*. This study aimed to describe the consequences of a point mutation in *Pdia6* on β-cell development and function.

**Methods:**

We generated an ENU mouse model carrying a missense mutation (Phe175Ser) in the second thioredoxin domain of the *Pdia6* gene. Using biochemical and molecular tools, we determined the effects of the mutation on the β-cell development at embryonic day (E)18.5 and β-cell identity as well as function at postnatal stages.

**Results:**

Mice homozygous for the Phe175Ser (F175S) mutation were mildly hyperglycemic at weaning and subsequently became hypoinsulinemic and overtly diabetic at the adult stage. Although no developmental phenotype was detected during embryogenesis, mutant mice displayed reduced insulin-expressing β-cells at P14 and P21 without any changes in the rate of cell death and proliferation. Further analysis revealed an increase in BiP and the PDI family member PDIA4, but without any concomitant apoptosis and cell death. Instead, the expression of prominent markers of β-cell maturation and function, such as *Ins2*, *Mafa*, and *Slc2a2*, along with increased expression of α-cell markers, *Mafb*, and glucagon was observed in adult mice, suggesting loss of β-cell identity.

**Conclusions:**

The results demonstrate that a global *Pdia6* mutation renders mice hypoinsulinemic and hyperglycemic. This occurs due to the loss of pancreatic β-cell function and identity, suggesting a critical role of PDIA6 specifically for β-cells.

## Highlights

• Mutation in the second thioredoxin domain (F175S) of PDIA6 does not affect pancreatic islet development.
• F175S mutation leads to development of neonatal diabetes in mice.
• Diabetic phenotype is due to severe lack of insulin and loss of β-cell identity.

## 1 Introduction

The two main forms of diabetes are type 1 and type 2 diabetes mellitus (T1DM and T2DM, respectively). While the former is characterized by an autoimmune-mediated β-cell death, the latter is more complex in nature, characterized by peripheral insulin resistance and β-cell dysfunction. The resultant hyperglycemia puts an undue burden on the small number of residual β-cells in T1DM, and the demands put forth by insulin resistance in T2DM augments a progressive loss of pancreatic β-cell identity and function.

Pancreatic β-cells generally have a high turnover of proteins, especially that of insulin. Protein disulfide isomerases (PDIs) are highly conserved ER-resident enzymes with oxidoreductase and isomerase activities, which are thought to facilitate the three essential disulfide bonds of proinsulin [1]. A less characterized member of this family is the protein disulfide isomerase associated 6 (PDIA6) [2], which contains two thioredoxin domains and is suggested to play a role in the folding of disulfide-bonded proteins [[3], [4], [5]].

Pancreatic β-cells are particularly predisposed to misfolding and subsequent ER stress even under physiological conditions to continually translate and package insulin protein and meet the metabolic demand [6]. Indeed, *in vitro* studies have reported an association of PDIA6 with misfolded proinsulin, suggesting its possible role in clearing misfolded protein [7]. Moreover, upon sustained ER stress, the unfolded protein response (UPR) is activated, which may eventually trigger the apoptotic pathway [6,8]. PDIA6 was found to interact with the ER stress sensors IRE1α and PERK, and loss of PDIA6 can reduce the expression of *Ins1* and *Ins2* transcripts via persistent IRE1α activity [3,4,9], indicating an indirect influence of PDIA6 on β-cell function *in vitro*. The evidence from these *in vitro* studies collectively implicates a role of PDIA6 in β-cell function and development of diabetes, which is further supported by a recent study that reported dysregulation of *Pdia6* in a T1DM model [10].

Here, we sought to explore the *in vivo* impact of a *Pdia6* point mutation on β-cell development and function as well as their overall metabolic effects. To this end, we generated a mouse model by a systematic random mutagenesis project using *N*-ethyl-*N*-nitrosourea (ENU) as the mutagen [[11], [12], [13]]. We isolated a recessive point mutation in the *Pdia6* gene leading to a Phe175Ser (F175S) exchange in the second thioredoxin domain. Homozygous mutant mice displayed reduced Mendelian ratio, suggesting pre-natal lethality. The surviving pups displayed reduced weight and rapidly developed hyperglycemia due to severe lack of insulin. Analysis of adult pancreatic islets revealed reduced β-cell markers and increased α-cell markers, which were in concert with loss of β-cell identity. Hence, this study demonstrates that although PDIA6 is not required for β-cell development, it is essential for the maintenance of pancreatic β-cell identity.

## 2 Materials and methods

### 2.1 *N*-ethyl-nitroso-*N*-urea (ENU) mutagenesis and mice

ENU mutagenesis was performed using the pure inbred C3HeB/FeJ mouse strain purchased originally from the Jackson Laboratory (Bar Harbor, Maine) as already described [11]. The c.524T > C mutation in *Pdia6* was isolated by candidate gene analysis of mutant mouse lines by exome sequencing, leading to the official name of the mouse line Pdia6^F175SMhda^. The mice were housed and handled according to the recommendations of the Directive 2010/63/EU; husbandry was in open type II or IVC cage systems enriched by bedding material, nestlets, and mouse houses. The state ethics committee and government of Upper Bavaria approved all animal studies (Gz. 55.2-1-54-2532-126-11 and 55.2-1-54-2532-144-10). Because of the reduced Mendelian ratio of homozygous offspring, mice were not separated based on sex and analyzed together.

### 2.2 Metabolic studies

Weekly blood glucose and body weight measurements were carried out at *ad libitum* fed state using Akku-Check (Roche) from tail blood.

### 2.3 Exome sequencing of the Pdia6^F175S^ point mutation

DNA extraction from spleens was performed using ProteinaseK, RNaseA, cell lysis solution, protein precipitation solution and DNA hydration solution according to the manufacturer's instructions (Qiagen). Exome sequencing was performed as described elsewhere [14].

### 2.4 Immunohistochemistry

Embryonic and P14 pancreata were dissected and fixed in 4% PFA in PBS for 2 h and-overnight at 4 °C, respectively. The tissues were merged in 7.5%, 15%, and 30% sucrose-PBS solutions at RT for 2 h at each step. They were then embedded in a cryoblock using tissue-freezing medium (Leica 14020108926), and sections of 20 μm thickness were cut. Next, the samples were permeabilized (0.1% Triton, 0.1 M Glycine) for 15 min and incubated in a blocking solution (10% FCS, 3% donkey serum, 0.1% BSA, and 0.1% Tween-20 in PBS) for 1 h at room temperature. Then, the primary antibodies (listed below) diluted in blocking solution were added to the samples and incubated overnight at 4 °C. After washing with PBST, they were stained with secondary antibodies (listed below) diluted in the blocking solution for 3–5 hs at room temperature. The samples were then incubated with 4’, 6-diamidin-2-phenylindol (DAPI), followed by washing with PBST and embedding in a commercial medium (Life Tech., ProLong Gold). All images were obtained with a Leica microscope of the type DMI 6000 using LAS AF software. Images were analyzed using LAS AF and ImageJ software programs.

P21 and adult pancreata were fixed in 4% neutral buffered formalin for 24 h and embedded in paraffin. Pancreatic tissue was cut into 7-μm serial sections with 3–4 sections pulled on SuperFrost® Plus slides (Menzel-Gläser). Sections were deparaffinized in xylene and rehydrated in descending alcohol concentrations. Next, heat-induced antigen retrieval was performed using Tris–EDTA (0.05% Tween-20, pH 9.0). Sections were then blocked in PBS solution with 1% BSA +5% horse serum +0.3% Triton for 2 h. Next, primary antibodies were added and incubated overnight at 4 °C. Following washing steps in PBST, secondary antibodies were added for 90 min at RT. After several washing steps, mounting medium (Vector Laboratories) was applied on the sections. Images were acquired using Zeiss Axio Imager M2 (fluorescent) and Zeiss LSM 880 (confocal). Images were processed and analyzed using ZEN 3.0 blue (Zeiss), Fiji and Definiens software (AstraZeneca). The antibodies used are provided below.

**Table:** 

Primary Antibody	Host	Working dilution	Company	Catalogue number
Chromogranin A	Rabbit	1:200	Abcam	ab15160
Cleaved caspase-3	Rabbit	1:300	Cell Signaling	9664
Glucagon	Guinea pig	1:3000	Takara	M182
Insulin	Guinea Pig	1:200	DAKO	A0564
Insulin	Rabbit	1:800	Cell Signaling	3014
Ki67	Rabbit	1:300	Abcam	ab15580
Nkx6-1	Goat	1:200	R&D Systems	AF5857-SP
Pdx1	Rabbit	1:300	Cell Signaling	5679
Proinsulin	Rabbit	1:200	DSHB	GS-9A8
Somatostatin	Rat	1:300	Invitrogen	MA5-16987
Urocortin 3	Rabbit	1:300	Phoenix Pharmaceuticals	H-019-29
Secondary Antibody	Host	Working dilution	Company	Catalogue number
AlexaFluor®488 anti-guinea pig	Goat	1:500	Invitrogen	A11073
AlexaFluor®488 anti-rabbit	Donkey	1:800	Invitrogen	A21206
AlexaFluor®555 anti-rabbit	Donkey	1:800	Invitrogen	A31572
AlexaFluor®555 anti-goat	Donkey	1:800	Invitrogen	A21432
AlexaFluor®546 phalloidin	–	1:200	Invitrogen	A22283
Cy3 anti-rat	Donkey	1:800	Dianova	712-165-153
AlexaFluor® 594 anti-rabbit	Donkey	1:500	Invitrogen	A21207
AlexaFluor® 594 anti-mouse	Donkey	1:500	Invitrogen	A21203
AlexaFluor® 488 anti-rabbit	Donkey	1:500	Invitrogen	A21206
AlexaFluor® 647 anti-goat	Donkey	1:500	Invitrogen	A21447
AlexaFluor® 647 anti-rat	Donkey	1:800	Dianova	712-605-150
AlexaFluor® 649 anti-guinea pig	Donkey	1:800	Dianova	706-495-148
AlexaFluor® 750-conjugated anti-rabbit	Goat	1:500	Invitrogen	A21039
DAPI	N/A	1:1000	Sigma–Aldrich	D9542

### 2.5 Protein extraction and western blots

Proteins from pancreatic tissue were extracted by suspending them in ice-cold RIPA Lysis and Extraction Buffer (Thermo Fisher Scientific) supplemented with 1x cOmplete® Mini Protease Inhibitor Cocktail (Roche) and PhosSTOP™ (Roche). Protein concentrations were determined using the Pierce BCA Protein Assay Kit (Thermo Fisher Scientific) according to the manufacturer's instructions. Next, 40 μg of protein sample was loaded onto a 10% SDS-polyacrylamide gel (Bio-Rad). Protein was then transferred to a nitrocellulose membrane (Thermo Fisher Scientific). The membrane was blocked with Odyssey® Blocking Buffer (TBS) (LI-COR) and incubated overnight at 4 °C with primary antibodies and secondary antibodies for 45 min at RT in the same buffer. For fluorescent detection of proteins, Odyssey Infrared Imaging System and Odyssey® software (LI-COR) were used. Densitometric quantification of western blot images was performed using Image Studio Lite version 5.2 (LI-COR) and expressed as relative fluorescence intensity. The used antibodies are provided below.

**Table:** 

Primary antibody	Host	Working dilution	Company	Catalogue number
Pdia6	Rabbit	1:1000	Proteintech	18233-1-AP
Pdia1	Rabbit	1:1000	Proteintech	11245-1-AP
Pdia4	Rabbit	1:1000	Proteintech	14712-1-AP
BiP	Rabbit	1:1000	Proteintech	11587-1-AP
IRE1A	Rabbit	1:1000	Cell Signaling	#3294
P-IRE1A	Rabbit	1:1000	Novus	NB100-2323
Alpha tubulin	Mouse	1:5000	Abcam	ab7291
Secondary antibody	Host	Working dilution	Company	Catalogue number
IRDye® 800CW anti-Rabbit	Goat	1:10,000	LI-COR	926–32211
IRDye® 680RD anti-Mouse	Goat	1:10,000	LI-COR	926–68070

### 2.6 Islet isolation and insulin measurements

Islet isolation was performed by digesting pancreatic tissue with collagenase P (Roche) solution and subsequently obtaining the islets in a gradient using Optiprep (Sigma). The islets were kept overnight in RPMI 1640 medium (Lonza) supplemented with 10% fetal bovine serum and 11 mM glucose. To obtain total islet protein content, islets were lysed in 70% acid-ethanol. Islet hormone content was measured using the mouse insulin ELISA kit (Mercodia), the glucagon ELISA kit (Mercodia), or the proinsulin ELISA kit (Mercodia) according to the manufacturer's instructions.

### 2.7 RNA isolation and qRT-PCR

Total RNA was isolated using the RNeasy Plus Micro kit (Qiagen), including digestion of the remaining genomic DNA, according to the manufacturer's instructions. qRT-PCR was used for the relative quantification of genes in islet cDNA samples by using the QuantiFast SYBR Green kit (Qiagen) according to the manufacturer's instruction. The results were determined as described elsewhere [15], and relative gene expression levels were normalized to those of housekeeping genes *Rpl13a* and *Ubc* by using the primer pairs given below.

**Table:** 

Gene	Forward sequence	Reverse sequence
*Rpl13a*	TGAAGCCTACCAGAAAGTTTGC	GCCTGTTTCCGTAACCTCAA
*Ubc*	AGCCCAGTGTTACCACCAAG	ACCCAAGAACAAGCACAAGG
*Slc2a2*	GGGGACAAACTTGGAAGGAT	TGAGGCCAGCAATCTGACTA
*Ins2*	CAGCAAGCAGGAAGCCTATC	GCTCCAGTTGTGCCACTTGT
*Pdia6*	ACACTGCAAAAACCTGGAGC	GTCAGATCTCGTCCGACCAC
*Gcg*	AGGCTCACAAGGCAGAAAAA	CAATGTTGTTCCGGTTCCTC
*Mafa*	CAGCAGCGGCACATTCTG	GCCCGCCAACTTCTCGTAT
*Mafb*	CTTCACGTCGAACTTGAGAAGG	TAGCGATGGCCGCGGAG
*Brn4*	CGAGAACACGTTGCCATAC	CCAACCTCTGATGAGTTGGAA
*Neurog3*	GTCGGGAGAACTAGGATGGC	GGAGCAGTCCCTAGGTATG

### 2.8 Re-analysis of healthy and mSTZ-diabetic mice

Processed, normalized, and annotated single cell RNA sequencing data were downloaded from GEO (accession number GSE132188). The original data contained cells from isolated pancreatic islets from seven different treatment groups [16]. For the scope of this manuscript, we re-analyzed a subset of the data that contained endocrine islets from healthy control mice and streptozotocin-treated (mSTZ) diabetic mice according to the clustering in Figure 4 of the original publication [16]. We used only mono-hormonal cells for the analysis presented in this manuscript.

**Figure 1:**
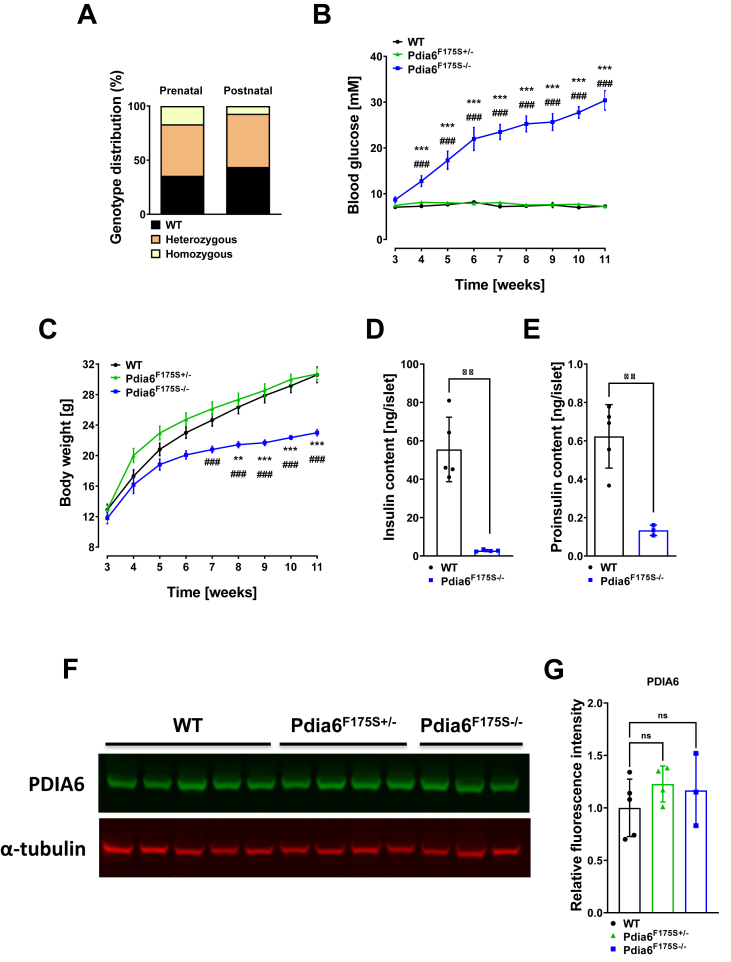
**F175S mutation leads to hyperglycemia and hypoinsulinemia.** (A) Graph displaying Mendelian ratio in mice acquired by heterozygous mating at prenatal (E18.5, 21 litters) and postnatal (P14, 8 litters) stages. **(B)** Weekly *ad libitum* blood glucose levels and **(C)** body weight in wild-type (WT), heterozygous (Pdia6^F175S+/-^), and homozygous (Pdia6^F175S−/−^) mice. n = 6–12. Whole islet content of **(D)** insulin and **(E)** proinsulin in isolated islets of WT and Pdia6^F175S−/−^ mice. n = 3–5. ∼15-week-old mice were used for this experiment; **(F)** Representative western blot image and **(G)** quantification of PDIA6 expression in the pancreatic tissue. n = 3–5. Three-week-old mice were used for this experiment. Error bars display ±SEM in B–C and ±SD in D-G. Differences were considered statistically significant at p < 0.05 using two-tailed Student's *t* test, one-way or two-way ANOVA with Bonferroni *post hoc* test. ∗ = Pdia6^F175S−/−^ vs. WT, # = Pdia6^F175S+/-^ vs. Pdia6^F175S−/−^ (∗*p* < 0.05, ∗∗*p* < 0.01, ∗∗∗, ###*p* < 0.001).

**Figure 2:**
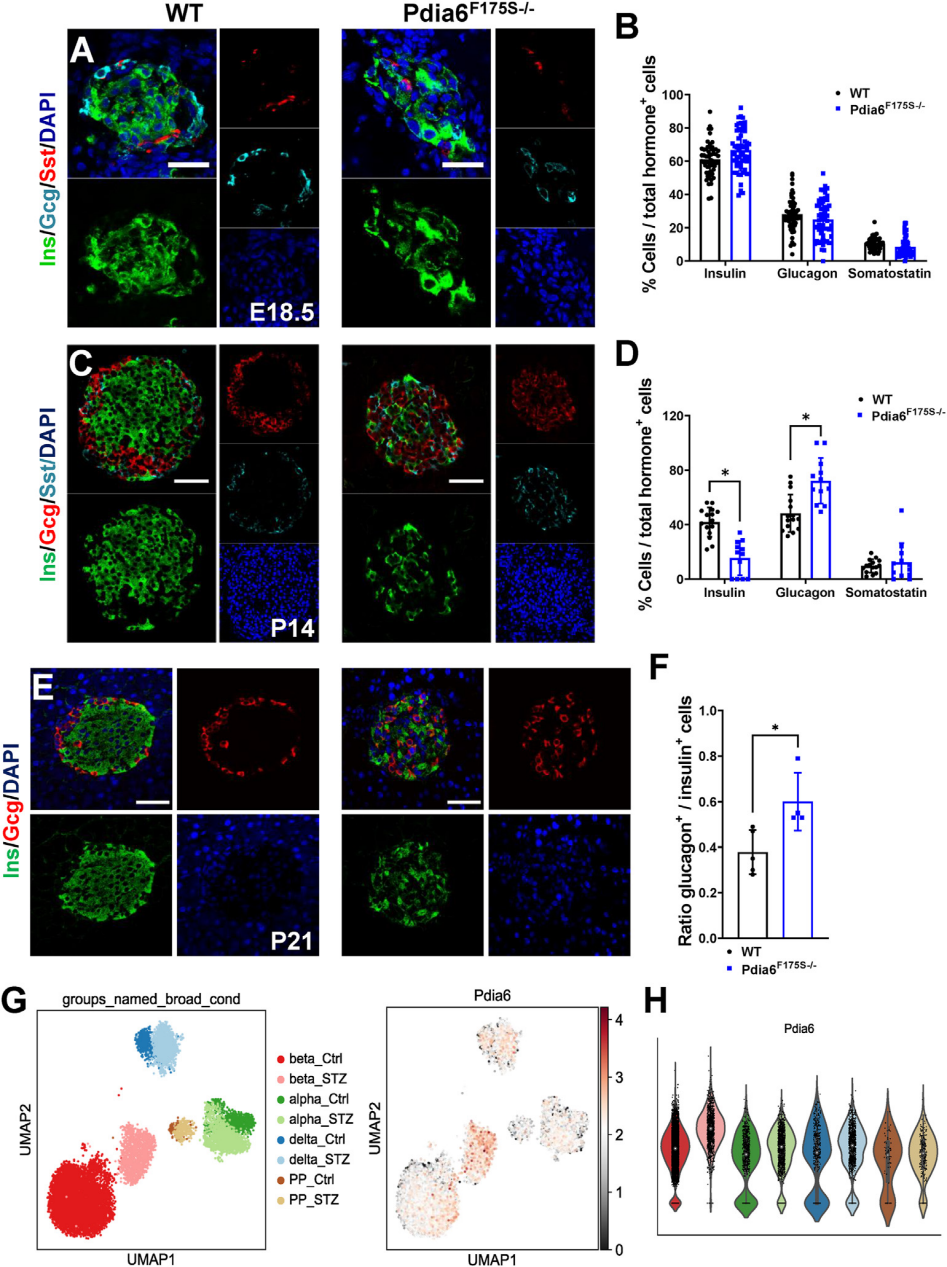
***Pdia6* point mutation results in a specific loss of β-cells at postnatal stages.** (A) Representative immunofluorescence images of insulin (green), glucagon (cyan), and somatostatin (red) in WT and Pdia6^F175S−/−^ mice at E18.5 and **(B)** quantification thereof. n = 3. Scale bar 30 μm. **(C)** Representative immunofluorescence images of insulin (green), glucagon (red), and somatostatin (cyan) in WT and Pdia6^F175S−/−^ mice at P14 and **(D)** quantification thereof. n = 3. **(E)** Representative immunofluorescence images of insulin (green) and glucagon (red) in WT and Pdia6^F175S−/−^ mice at P21. **(F)** Glucagon-to-insulin positive cell ratio at P21. n = 4–5. Scale bar = 50 μm. **(G)** UMAP plot from single cell RNA-Seq data [16] on sorted islet cells in control and STZ-treated groups. **(H)** Violin plot displaying increased *Pdia6* expression specifically in STZ-treated β-cells. Error bars display ±SD. Differences were considered statistically significant at *p* < 0.05 using a two-tailed Student's *t* test (∗*p* < 0.05, ∗∗*p* < 0.01, ∗∗∗*p* < 0.001).

**Figure 3:**
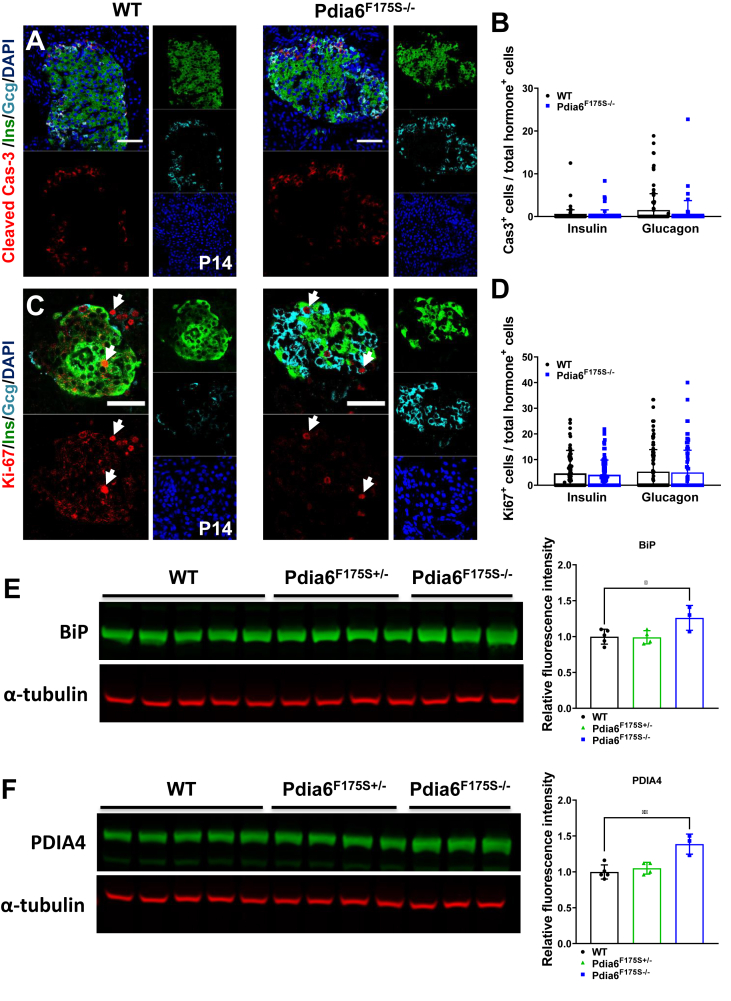
**No change in β-cell apoptosis or proliferation in mutant mice.** (A) Representative immunofluorescence images of apoptotic insulin and glucagon positive cells by cleaved caspase 3 (red) and **(B)** quantification thereof in WT and Pdia6^F175S−/−^ mice at P14. **(C)** Representative immunofluorescence images of proliferative insulin and glucagon positive cells by Ki67 (red) and **(D)** quantification thereof in WT and Pdia6^F175S−/−^ mice at P14. n = 3. Scale bar = 50 μm. Western blot images and quantifications of relative protein content of **(E)** BiP, and **(F)** PDIA4 in pancreatic tissue of P21 mice. All values are normalized to the loading control α-tubulin. n = 3–5. Error bars display ±SD. Differences were considered statistically significant at *p* < 0.05 using a two-tailed Student's *t* test (∗*p* < 0.05, ∗∗∗*p* < 0.001). n.s = non-significant.

**Figure 4:**
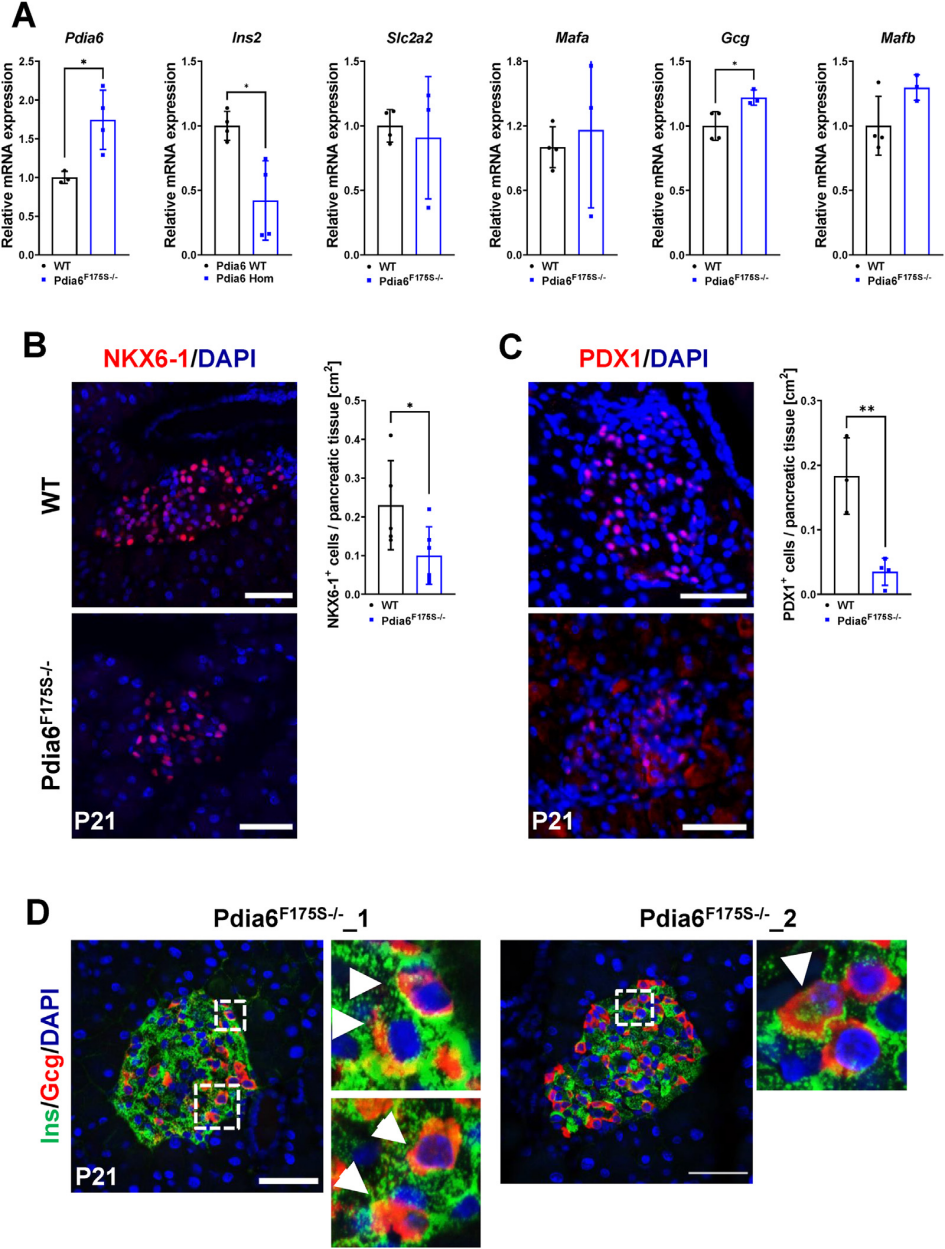
**Point mutation in *Pdia6* leads to loss of β-cell identity.** (A) mRNA expression of several α- and β-cell markers. n = 3–4. **(B)** Representative immunofluorescence images and quantification of β-cell markers NKX6-1 and **(C)** PDX1. n = 3–6. **(D)** Representative immunofluorescence images displaying presence of insulin and glucagon double positive cells in Pdia6^F175S−/−^ mutant mice. Mice at P21 were used in all experiments. Scale bar = 50 μm. Error bars display ±SD. Differences were considered statistically significant at *p* < 0.05 using a two-tailed Student's *t* test (∗*p* < 0.05, ∗∗∗*p* < 0.001).

### 2.9 Statistical analysis

Statistical analysis was achieved using GraphPad Prism 9.0 and applied using two-tailed Student's *t* test, one-way or two-way ANOVA followed by the *post hoc* Bonferroni test. A value of *p* < 0.05 was considered significant, and all results are described as means and standard of error of mean (±SEM) or standard deviation (±SD), as indicated in the legends. Sample number designated by “n” represents number of individual mice. Data points display either individual islets or means of islets on pancreatic sections for the quantification of immunofluorescence images at E18.5 and P14.

## 3 Results

### 3.1 F175S mutation leads to hyperglycemia and hypoinsulinemia

We generated an ENU mutant mouse line where a T to C exchange at position 524 in exon 6 of the *Pdia6* gene produces an amino acid exchange at phenylalanine 175 to a serine residue (F175S) (c.524T > C) at a conserved sequence between humans and mice (Fig. S1A). The phenylalanine at position 175 (F175, magenta) resides in the second thioredoxin domain and drives the main interactions between the alpha helix 1 and the hydrophobic pocket, which is facilitated by V179 (Fig. S1B), conferring its catalytic property [5]. Following heterozygous intercrosses, we observed a reduced Mendelian ratio for homozygous *Pdia6* mutants (Pdia6^F175S−/−^) at embryonic day (E)18.5, which is further declined at postnatal stages (Figure 1A), indicating reduced survival in the homozygous state.

Pdia6^F175S−/−^ pups depicted mild increase in blood glucose levels at weaning age, progressing to severe hyperglycemia in the next following weeks (Figure 1B). Pdia6^F175S−/−^ mice also displayed significantly reduced body weight gain over time on a chow diet (Figure 1C). Plasma insulin levels of Pdia6^F175S−/−^ mice were below the detection threshold of the used insulin ELISA kit. Measurement of the insulin content in isolated islets from Pdia6^F175S−/−^ mice revealed over 80% reduction compared to that of wild-type (WT) mice (Figure 1D), corresponding to the undetectable amounts of plasma insulin and acute hyperglycemia in these animals. PDIA6 has been suggested to play a role in the correct folding of proinsulin into mature insulin protein [7]. Thus, we stained pancreatic sections for proinsulin and found little amount of protein expression in mutant mice (Fig. S1C). Accordingly, proinsulin levels were also found to be reduced in Pdia6^F175S−/−^ islets (Figure 1E). Next, we investigated whether the prevailing phenotype was due to loss of the PDIA6 protein. Interestingly, we found no significant change in the amount of protein in the pancreatic tissue of mutant mice (Figure 1F–G), indicating that the F175S mutation does not lead to a loss of PDIA6 protein. Taken together, a point mutation in the second thioredoxin domain of PDIA6 renders mice diabetic.

### *3.2* *Pdia6* point mutation results in a specific loss of β-cells at postnatal stages

Pdia6^F175S−/−^ mice showed higher blood glucose levels at weaning, which suggested that β-cell failure arises at an earlier time point either during embryonic or early postnatal development. Thus, we carried out immunostaining of pancreatic sections at E18.5 and found no obvious difference in islet composition (Figure 2A–B). Next, we wondered whether the reduced insulin levels resulted from distortion in early postnatal development. To this end, we analyzed the islet cell composition at postnatal day (P) 14. While the number of δ-cells was comparable between the groups, we found a relative increase in α-cells concomitant with a decrease in β-cells in islets from Pdia6^F175S−/−^ mice (Figure 2C–D). Consistently, at P21 Pdia6^F175S−/−^ mice displayed dramatically reduced insulin staining and centrally scattered α-cells, effectively increasing α-to β-cell ratio (Figure 2E–F). These data suggest that although endocrine pancreatic development is unaffected, loss of insulin positive β-cells occurs postnatally, before the advent of hyperglycemia.

To further support that this *Pdia6* mutation results in a β-cell specific phenotype, we analyzed the expression of *Pdia6* in different endocrine cell types at single cell levels by using our previously reported dataset [16]. We found a comparable expression level of this gene in α-, β-, δ-, and PP-cells in healthy adult mouse islets. However, in streptozotocin (STZ)-treated diabetic animals, a specific increase in *Pdia6* expression level was observed in β- but not in non-β endocrine cells (Figure 2G–H), suggesting that *Pdia6* is upregulated upon cytotoxic stress. These data suggest a critical function of *Pdia6* in β-cell homeostasis and upon cellular stress, supporting the findings that a mutation in this gene specifically impairs β-cell function.

### 3.3 No change in β-cell apoptosis or proliferation in mutant mice

To determine the cause of β-cell loss, we investigated the state of apoptosis in these mice. In keeping with a normal islet composition at E18.5, we did not observe any changes in apoptosis and proliferation between the groups (Figs. S2A–D). Interestingly, when we analyzed mice at P14, where we first observed changes in islet composition, we again did not observe any significant changes in the apoptotic and proliferative markers in homozygous mutant mice (Figure 3A–D). Additionally, we analyzed the expression of chromogranin A (ChgA) in mutant mice at P21 and did not find any significant changes between both groups, suggesting maintenance of the endocrine lineage and supporting the lack of apoptosis (Figs. S2E–F).

Because PDIA6 was shown to interact with proinsulin [7] and PDIs are involved in disulfide-bond formation [1], a *Pdia6* mutation may lead to insulin misfolding and subsequent ER stress. Therefore, we investigated the state of some intermediates of this pathway. We isolated protein from pancreatic tissue of P21 mice and carried out western blot analysis. We investigated protein levels of the ER chaperone BiP (encoded by *Hspa5*) as well as IRE1α, a direct interactor of PDIA6 [9]. We observed a significant increase in BiP levels in *Pdia6*^F175S−/−^ animals (Figure 3E), indicating the presence of ER stress. However, the levels of phosphorylated IRE1α remained comparable between the groups (Fig. S3A). In addition, we determined the protein levels of PDI family members PDIA1 and PDIA4 and observed normal expression of PDIA1 (Fig. S3B) but a significant increase in the protein level of PDIA4 in homozygous *Pdia6* mutant mice (Figure 3F), Thus, these data suggest that the F175S mutation in PDIA6 results in a modest increase in ER-stress without any cellular death via apoptosis.

### 3.4 Point mutation in *Pdia6* leads to loss of β-cell identity

To further explore how the number of β-cells is reduced in *Pdia6* mutants, we analyzed the expression of key β-cell genes using qPCR analysis on isolated islets from Pdia6^F175S−/−^ and WT mice at P21. Interestingly, mRNA levels of *Pdia6* were significantly upregulated in the islets of mutant mice (Figure 4A). We found a decrease in the expression levels of Insulin (*Ins2*), while comparable levels of two major β-cell maturation markers *Slc2a2* (encoding for the glucose transporter GLUT2) and *Mafa* were observed (Figure 4A). In contrast, the expression level of *Gcg* was increased, along with an increased tendency in the α-cell transcription factor *Mafb* (Figure 4A).

Next, we analyzed the expression of β-cell transcription factors NKX6-1 and PDX1 in pancreatic sections at P21. We observed a significant reduction in both NKX6-1- (Figure 4B) and PDX1-positive cells (Figure 4C) in mutant islets. Upon closer inspection, we detected endocrine cells positive for both insulin and glucagon in *Pdia6* mutant animals (Figure 4D), highlighting the appearance of polyhormonal cells. Altogether, these data suggest that mutation in *Pdia6*^F175S−/−^ results in loss of β-cell identity with concomitant upregulation of lineage-inappropriate α-cell markers.

Finally, we analyzed adult mice (12–15 weeks) and found that loss of β-cell identity was exacerbated with a significant decrease in the expression of *Mafa* and *Slc2a2* accompanied with an increase in *Mafb,* α-cell specification marker *Brn4* as well as *Neurog3*, strongly indicating loss of β-cell identity [17] (Figs. S4A–B). This increase was reflected in glucagon content in isolated islets of mutant mice (Fig. S4C). Accordingly, the expression of GLUT2 and insulin was found to be dramatically reduced (Fig. S4D). Thus, the data argue for a progressive loss of β-cell identity in *Pdia6* mutant mice, which initiates at around weaning and becomes prominent in adult mice.

## 4 Discussion

After translation and translocation of proinsulin to the ER, PDIs are thought to facilitate the formation of the three essential disulfide bonds of proinsulin and, as such, play an important role in insulin synthesis [[1], [18]]. The β-cells of the pancreas rely heavily on a highly efficient and functional ER to meet the metabolic demand of insulin production. A derangement of ER homeostasis may result in β-cell dysfunction**.** In the present study, we generated a mouse model that carries the F175S mutation in PDIA6, a member of the PDI family, to study the effects on β-cell function. Homozygous *Pdia6* mutant mice show normal islet development and β-cell maturation. However, these mice postnatally progress to a hyperglycemic state rapidly due to loss of insulin production. Surprisingly, the mutation did not lead to a loss of *Pdia6* expression nor did we observe any change in PDIA6 protein expression. Nevertheless, proinsulin and insulin content of islets were decreased, indicating that a loss of PDIA6 function may have a greater effect than loss of PDIA6 protein *per se*, as exemplified by a study showing that the absence of PDIA6 in the INS-1 cell line did not alter proinsulin folding [3]. This is in agreement with our model that shows a decrease in *Ins2* expression rather than an accumulation of proinsulin. In contrast, the absence of PDIA1, the most abundant ER oxidoreductase, indeed lead to altered proinsulin folding [19]. This suggests that although PDIA6 may aid in the clearance of misfolded proinsulin, it probably regulates insulin production via other mechanisms. Indeed, some evidence for an indirect role of PDIA6 in this context was reported where lack of PDIA6 lead to a decrease in *Ins1* & *Ins2* expression via IRE1a [9]. *Pdia6* mutant mice showed a mild reduction in the expression of the *Ins2* transcript at P21 but massive reduction in β-cell markers concomitant with an increase in α-cell markers at the adult stage, strongly suggesting a progressive loss of β-cell identity. Simultaneously, we observed significantly increased BiP protein in Pdia6^F175S−/−^ mice, indicating the presence of ER-stress. The expression of PDIA1, however, was normal, whereas the PDIA4 protein was increased in mutants. However, we did not observe any increase in apoptosis, reduction in proliferation or change in neuroendocrine lineage marker ChgA, collectively pointing to the lack of β-cell death. Thus, the data indicate that the reduction in insulin is not due to increased β-cell death but rather due to the loss of β-cell identity, which may in part be exacerbated by hyperglycemia itself [20]. Our data support a paradigm where PDIA6 expression and its effects on insulin expression are more tightly linked with β-cell identity and homeostasis than previously appreciated. Likewise, the deletion of ER stress sensor proteins such as *Eif2ak3* in the pancreas and *Ire1α* in β-cells do not lead to increased β-cell death either [[21], [22]], but rather demonstrate a loss of β-cell identity. This is also echoed by the fact that dysregulated expression of several ER stress components, including that of *Pdia6*, have been reported to precede the development of T1DM [[10], [23]]. Hence, one can posit that manipulating β-cell identity and thereby restraining UPR, prior to the onset of diabetes, might be a viable therapeutic option [[22], [24]]. It would be interesting to test the efficacy of PDIA6 in this context.

*Pdia6* is ubiquitously expressed in both human and mouse tissues [25]. Thus, our *Pdia6* model with a global mutation represents a complex phenotype that might have implications for organ crosstalk and may even involve central control of metabolism, warranting further investigation. However, the present study, including two other studies [[26], [27]], argue for a β-cell dysfunction as at least one of the primary defects of a non-functional PDIA6. Choi et al. investigated a compound mouse model with a point mutation in the first thioredoxin domain of *Pdia6* and reported a loss of the PDIA6 protein [27]. The *Pdia6* model in the present study has a mutation in the second thioredoxin domain, without the loss of the PDIA6 protein, suggesting divergence in the functional aspects of the two domains [28]. The main interactions between the alpha helix 1 and the hydrophobic pocket are facilitated by phenylalanine (F175, magenta) and V179 (Fig. S1B). Mutation of F175 to serine most likely results in a misfolded or displaced alpha helix 1. This could result either in an inactive conformation or in unspecific aggregation due to a large hydrophobic area on the protein surface, suggesting presence of an inactive PDIA6 protein. Moreover, similarities between the two *Pdia6* mouse models with regard to the metabolic phenotype reinforce the importance of PDIA6 in β-cell function. This is further strengthened by a recent case study that reported a frameshift mutation in *PDIA6* in an infant as the cause of neonatal diabetes due to severely reduced insulin levels, among other developmental defects [26]. In line with this, Choi et al. failed to generate any homozygous mutants and a reduced Mendelian ratio of homozygous Pdia6^F175S−/−^ mice demonstrates the crucial role of a functional ER stress machinery during mammalian development and neonatal growth [[27], [29], [30], [31]], which requires further examination. Taken together, the phenotype of Pdia6^F175S−/−^ mice points to hallmarks of an early onset diabetic phenotype, signifying the contribution of PDIA6 in the maintenance of β-cell identity and the development of diabetes.

## Author contributions

NFC designed the study, performed experiments, analyzed and interpreted the data, and wrote the manuscript. ALA performed experiments, analyzed and interpreted data, contributed to the discussion, and reviewed the manuscript. ABP performed experiments, analyzed and interpreted the data, and reviewed the manuscript. SJS, AF, MR, and MTM performed experiments. BLD and SS generated the mouse line and reviewed the manuscript. MB, HL, and GP supervised and coordinated experiments. MB, GP, and MHdA conceived and designed the study, contributed to the interpretation of results, and critically reviewed the manuscript. MHdA is the guarantor of this work and, as such, had full access to all the data in the study and takes responsibility for the integrity of the data and the accuracy of the data analysis. All authors approved the final version of the manuscript.
