# Supplementary material for: A point mutation in the Pdia6 gene results in loss of pancreatic β-cell identity causing overt diabetes
Source: Mol Metab. 2021 Sep 4;54:101334. doi: 10.1016/j.molmet.2021.101334 (PMC8515296; doi:10.1016/j.molmet.2021.101334)
Supplement: Multimedia component 1 [file mmc1.pdf]

Figure S1

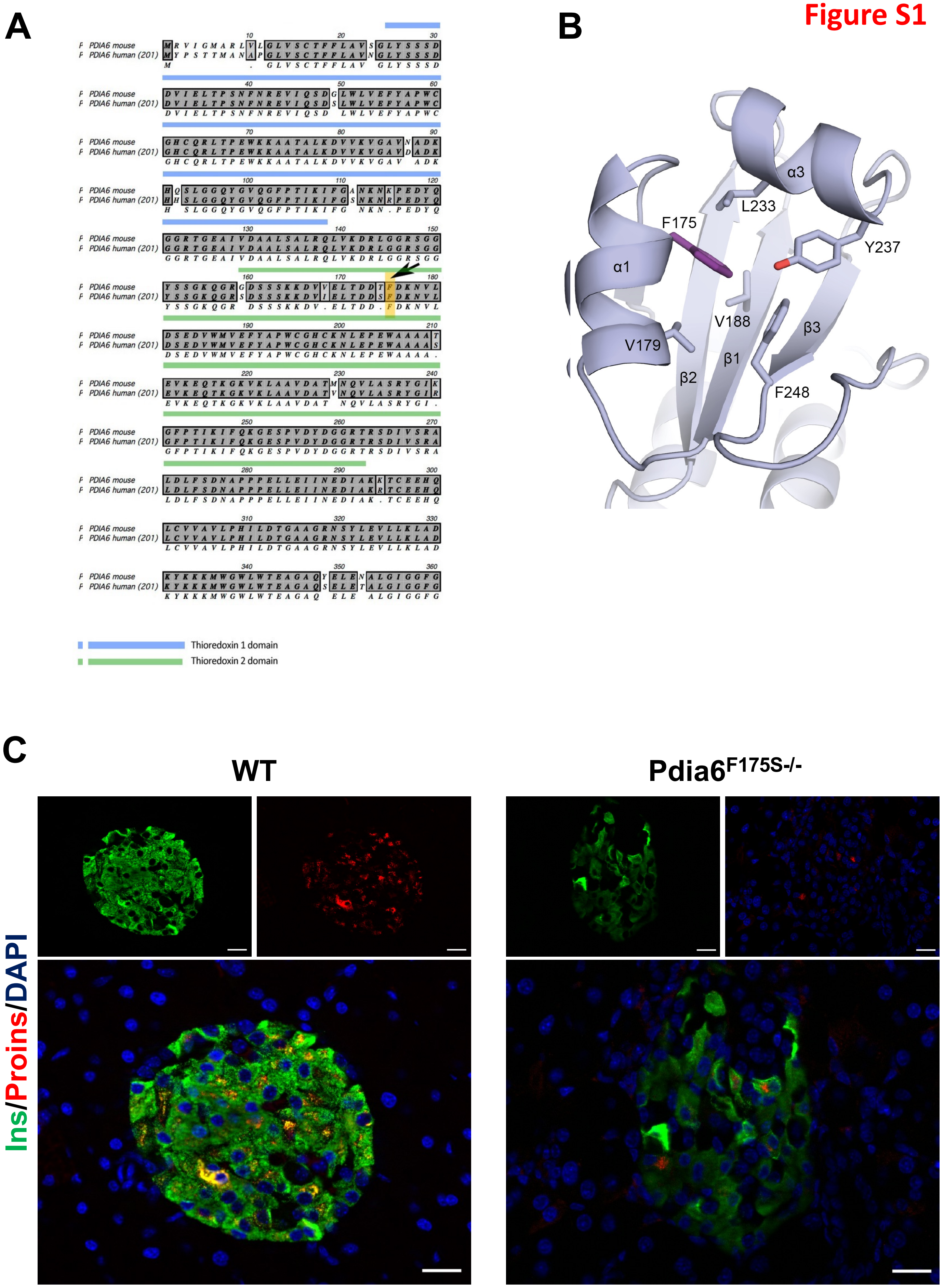
A
PDIA6 mouse
PDIA6 human (201)
Thioredoxin 1 domain
Thioredoxin 2 domain
B
α3
L233
F175
Y237
α1
V188
β3
V179
β1
β2
F248
C
WT
Pdia6F175S-/-
Ins/Proins/DAPI

Figure S2

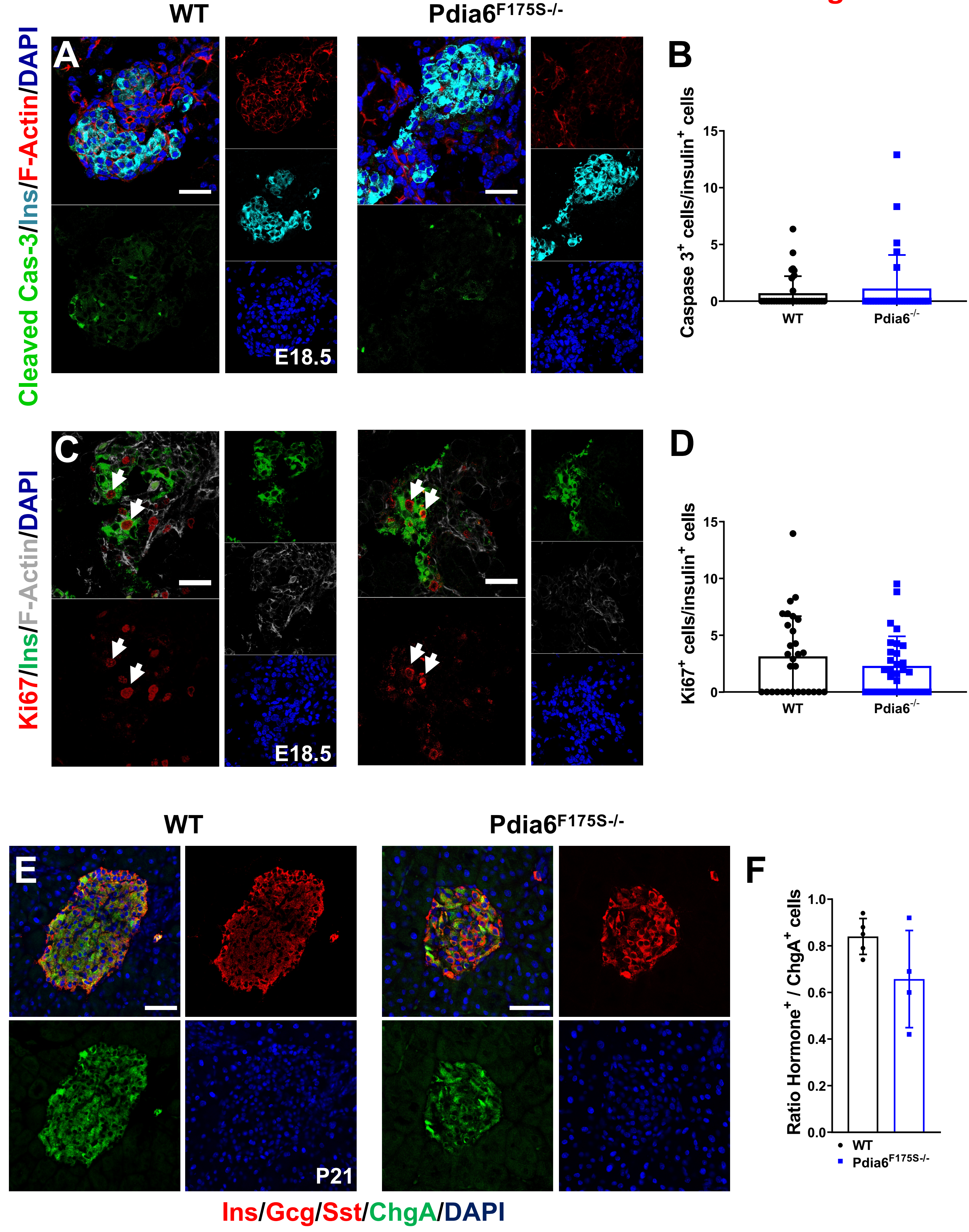
WT
Pdia6F175S-/-
A
Cleaved Cas-3/Ins/F-Actin/DAPI
E18.5
B
Caspase 3+ cells/insulin+ cells
15
10
5
0
WT
Pdia6-/-
C
Ki67/Ins/F-Actin/DAPI
E18.5
D
Ki67+ cells/insulin+ cells
15
10
5
0
WT
Pdia6-/-
WT
Pdia6F175S-/-
E
P21
Ins/Gcg/Sst/ChgA/DAPI
F
Ratio Hormone+ / ChgA+ cells
1.0
0.8
0.6
0.4
0.2
0.0
WT
Pdia6F175S-/-

Figure S3

A

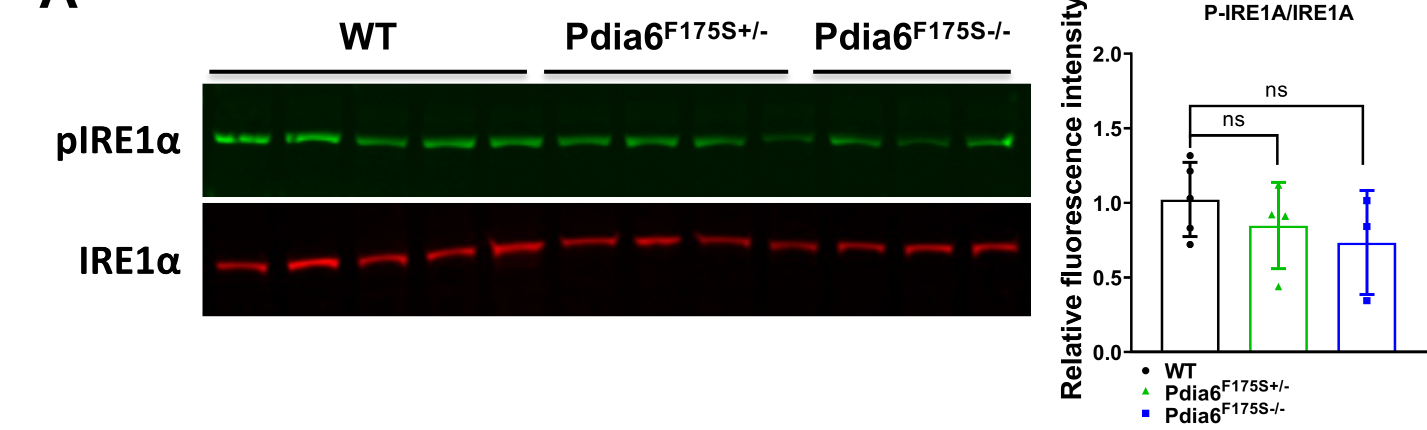
WT
Pdia6F175S+/-
Pdia6F175S-/-
pIRE1α
IRE1α
P-IRE1A/IRE1A
Relative fluorescence intensity
2.0
1.5
1.0
0.5
0.0
ns
ns
WT
Pdia6F175S+/-
Pdia6F175S-/-

B

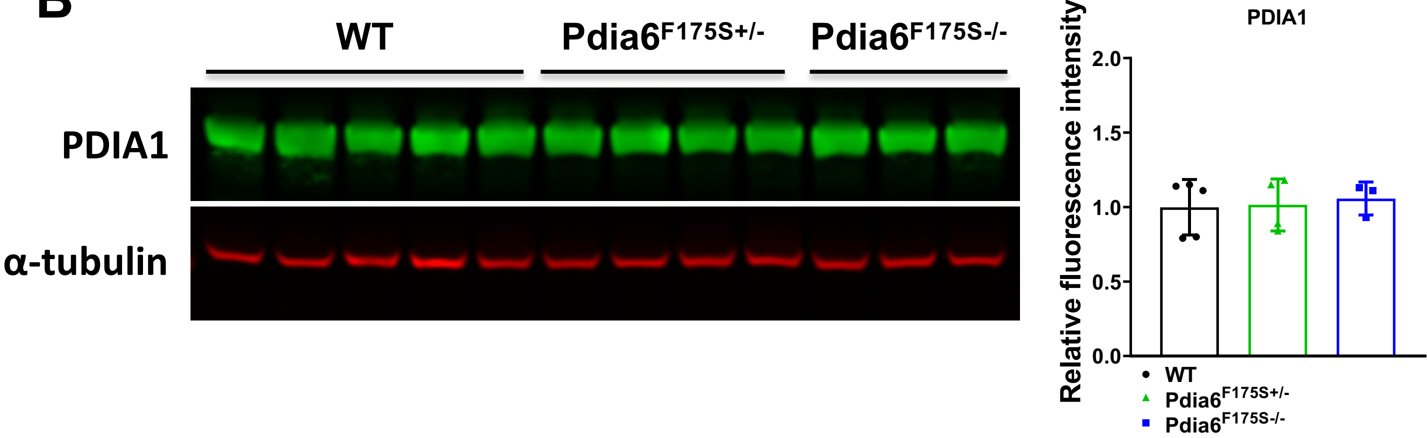
WT
Pdia6F175S+/-
Pdia6F175S-/-
PDIA1
α-tubulin
PDIA1
Relative fluorescence intensity
2.0
1.5
1.0
0.5
0.0
WT
Pdia6F175S+/-
Pdia6F175S-/-

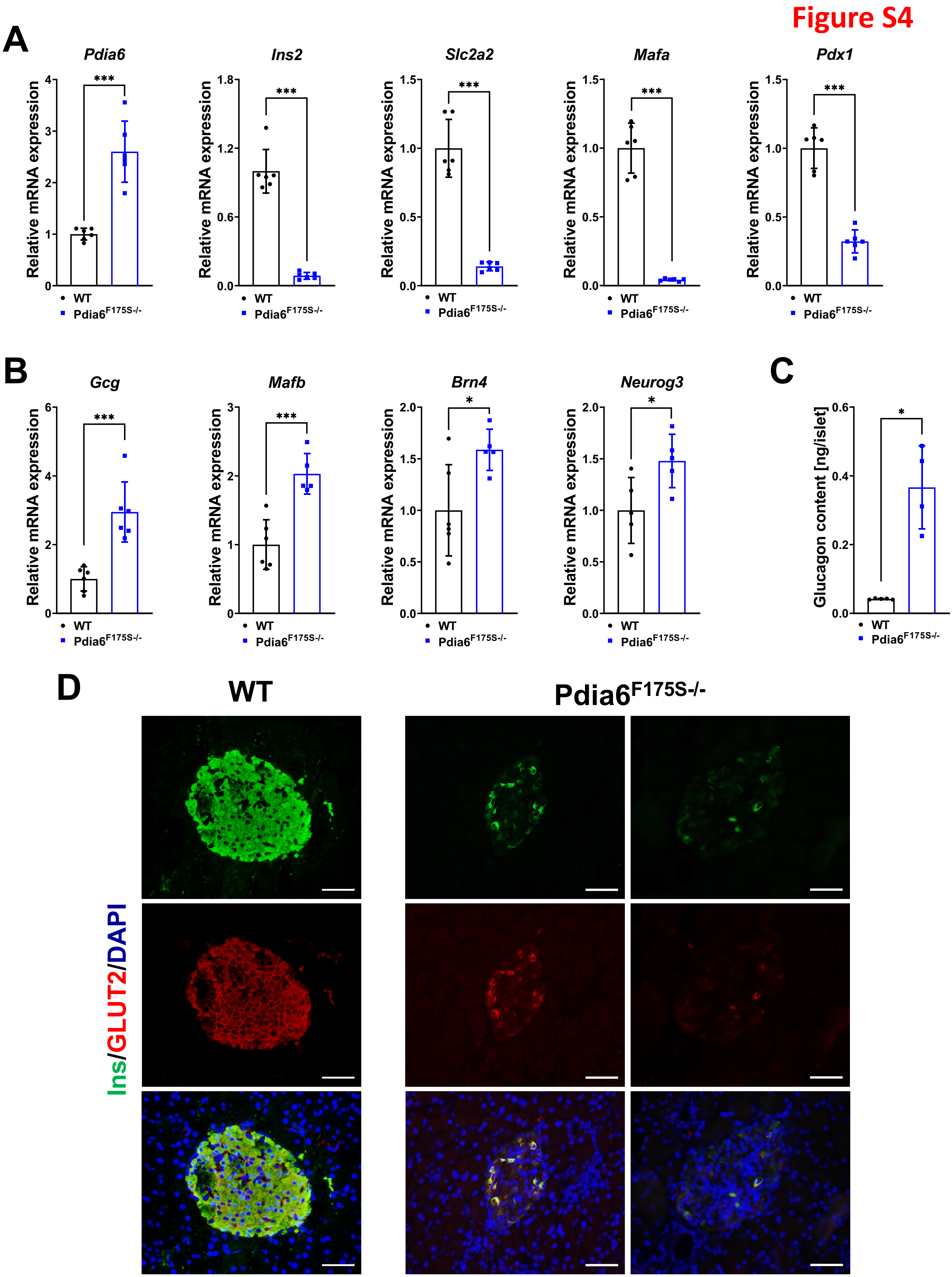
Figure S4
A
Pdia6
Ins2
Slc2a2
Mafa
Pdx1
Relative mRNA expression
***
WT
Pdia6F175S-/-
B
Gcg
Mafb
Brn4
Neurog3
*
C
Glucagon content [ng/islet]
D
WT
Pdia6F175S-/-
Ins/GLUT2/DAPI

Figure legends

Figure S1: (A) The human and mouse *Pdia6* genes are highly conserved. The black arrow points to phenylalanine at position 175 that is mutated in the *Pdia6* ENU mutant. **(B)** The image shows a detailed view of the second thioredoxin domain of PDIA6 (PDB: 1X5D) and displays the hydrophobic pocket (residues V188, L233, Y237 and F248) surrounding the phenylalanine (F175, displayed in magenta) where the F175S mutation lies. **(C)** Representative images of insulin (green) and proinsulin (red) expression in islets of 12-weeks-old WT and mutant mice. Scale bar 20 µm.

Figure S2: (A) Determination of apoptosis in insulin positive cells of Pdia6$^{F175S-/-}$ embryos (E18.5) and **(B)** quantification thereof. **(C)** Determination of proliferative insulin positive cells by Ki67 (red) immunostainings in WT and Pdia6$^{F175S-/-}$ at E18.5 and **(D)** quantification thereof. n=3. Scale bar 30 µm. **(E)** Representative immunofluorescence images of insulin, glucagon and somatostatin (all red) and ChgA (green). **(F)** Hormone-to-ChgA positive cell ratio in WT and Pdia6 $^{F175S-/-}$ at P21. n=4-5. Scale bare 50 µm. Error bars display ±SD.

Figure S3: Western blot images and quantifications of relative protein content of **(A)** pIRE1α/IRE1α and **(B)** PDIA1 in pancreatic tissue of P21 mice. All values are normalized to the loading control α-tubulin. n=3-5. Error bars display ±SD. No statistical differences were found using two-tailed Student's *t* test and one-way ANOVA with Bonferroni *post hoc* test.

Figure S4: mRNA expression of several **(A)** β-cell and **(B)** α-cell markers and *Neurog3* in islets. n=5-6. **(C)** Islet glucagon content in WT and Pdia6$^{F175S-/-}$ mice. n=4-5. **(D)** Representative immunofluorescence images of insulin (green) and GLUT2 (red) expression. 12-15-weeks-old mice were used for all experiments. Scale bar 50 µm. Error bars display ±SD. Differences were considered statistically significant at p<0.05 using a two-tailed Student's *t* test (*p<0.05, ***p<0.001).
